# Ethylene reshapes protein assemblies in the dark

**DOI:** 10.1093/plphys/kiaf605

**Published:** 2025-11-19

**Authors:** Nicola Trozzi, Alicja B Kunkowska

**Affiliations:** Assistant Features Editor, Plant Physiology, American Society of Plant Biologists; The Mechanobiology Laboratory, Department of Plant Molecular Biology, University of Lausanne, Lausanne CH-1015, Switzerland; Assistant Features Editor, Plant Physiology, American Society of Plant Biologists; PlantLab, Institute of Plant Sciences, Sant’Anna School of Advanced Studies, Pisa 56010, Italy

When seedlings push through the soil, they face shifting pressure and complete darkness. Mechanical resistance triggers the production of ethylene, a gaseous hormone that slows elongation and thickens the stem, helping the young plant safely push upward through dense soil (Qiao et al. 2012; Vandenbussche et al. 2012). Recently in *Plant Physiology*, Lee et al. (2025) examined the early protein-level responses that occur when dark-grown *Arabidopsis thaliana* hypocotyls are exposed to ethylene. Their study focused on the first 2 hours of treatment, a period in which ethylene rapidly suppresses elongation and promotes radial growth. By applying quantitative proteomics and co-fractionation mass spectrometry, the authors mapped how the proteome responds during this short transition. The work was designed to capture changes in protein abundance and complex organization that might explain how ethylene quickly redirects growth in darkness, indicating that ethylene shapes growth at the protein level while also working through established transcriptional pathways.

The authors used quantitative proteomics and co-fractionation mass spectrometry to study how ethylene changes both protein abundance and complex formation in etiolated hypocotyls, building on earlier approaches for mapping native protein complexes (Aryal et al. 2014; McBride et al. 2019). The classic “triple response,” defined by short hypocotyls, thick stems, and exaggerated hooks, was reproduced under controlled ethylene treatment, confirming that the experimental setup reflected normal physiology. Overall protein amounts changed little, but the distribution of proteins across size-exclusion fractions shifted strongly. Many proteins appeared in new high- or low-molecular-weight complexes, showing that ethylene reorganizes existing assemblies rather than triggering new synthesis. These fast rearrangements may depend on phosphorylation or proteolytic processing, both common ways to modify protein behavior in early signaling.

Many of the affected proteins were ribosomal or involved in translation, showing that ethylene reaches into the core of protein synthesis. Ribosomal subunits shifted between free and assembled forms, consistent with a brief pause in elongation. Proteins linked to ubiquitin–ligase complexes, vesicle transport, and redox balance changed their arrangement as well, processes that influence how cells grow and expand. Together, these shifts show that multiple cellular systems, including ribosomes, proteasomes, and trafficking components, alter their assembly states in a coordinated way during ethylene signaling. These changes took place within minutes, consistent with earlier work showing that ethylene can rapidly adjust growth and translation before new transcripts appear (Binder et al. 2004; Li et al. 2015).

A closer look at 3 proteins showed distinct remodeling patterns (Figure). DDB1-ASSOCIATED WD40 PROTEIN 1 (DWA1), a WD40-domain subunit of the CUL4 ubiquitin ligase, assembled into new high-mass complexes, which may redirect protein turnover during growth arrest. ASPARTIC PROTEASE IN A1 (APA1) shifted from single to multimeric forms, matching an increase in protein breakdown under ethylene treatment. After ethylene exposure, soluble fragments of the ATP-BINDING CASSETTE transporter ABCB5 appeared, suggesting that proteolytic cleavage released parts of the membrane protein into the soluble fraction. Seedlings lacking DWA1 or APA1 responded weakly to ethylene, linking these protein assemblies directly to growth control.

**Figure:**
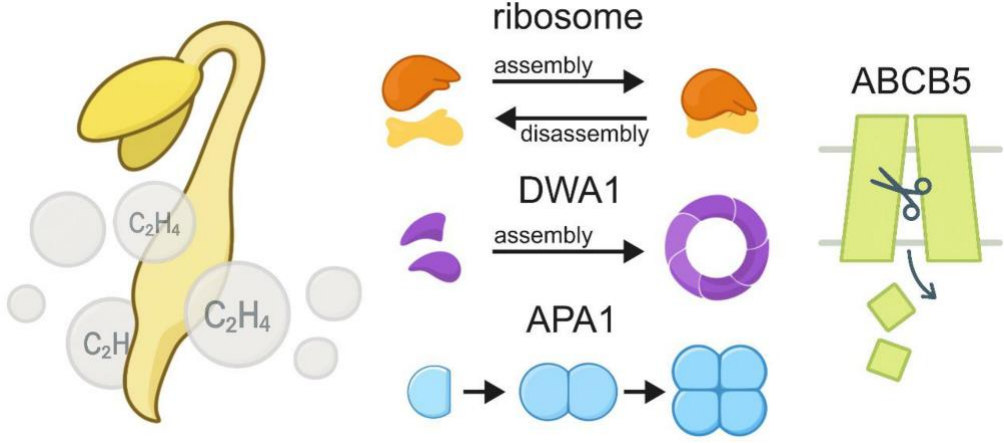
Ethylene reshapes protein assemblies in etiolated hypocotyls. Two hours of ethylene exposure triggers widespread remodeling of protein complexes in dark-grown *Arabidopsis* seedlings. Ribosomal subunits shift between assembled and monomeric states, while the ubiquitin-ligase subunit DWA1 and the vacuolar protease APA1 form larger complexes. The ABC transporter ABCB5 appears as soluble fragments following cleavage of membrane-bound forms. These molecular shifts occur within hours, before major transcriptional responses are detected, contributing to ethylene-induced inhibition of elongation and radial swelling of the hypocotyl.

All the results point to an ethylene response shaped by many post-translational steps. Proteins join and separate, and changes such as phosphorylation and proteolysis turn hormone signals into shifts in cell structure. The proteasome and ribosome, once seen as passive parts of the cell, now take an active role in adjusting growth through reversible protein links. Similar reorganizations happen in animal cells, showing a common way for cells to change quickly through protein-level control (Aebersold and Mann 2016). In plants, similar hormone-driven protein rearrangements have been described in other systems (Zeng et al. 2018), and earlier work suggested that multimerization can drive new functions in proteins (Lee and Szymanski 2021).

By combining proteomics, chromatography, and developmental assays, Lee and colleagues offer both a dataset and a new way to look at ethylene action. This work provides the first proteome-wide map of ethylene-dependent multimerization, a resource that reveals how protein assemblies reorganize within minutes of signaling. The hormone does more than change gene expression; it reorganizes the proteome's structure. Future studies that link co-fractionation with phosphoproteomics or live imaging could show how these molecular shifts lead to the visible changes in hypocotyl growth. The work expands our understanding of hormone responses, putting protein complex dynamics at the center of plant developmental control. The same approach may uncover equally fast protein rearrangements in responses to other hormones or environmental stress.

**Recent research articles in *Plant Physiology*:**

- Schaefer et al. (2023) showed that WAVE DAMPENED2 LIKE4 constrains temperature induced hyper elongation in light grown hypocotyl cells, linking cytoskeleton associated factors to hormone dependent growth control.
- Xu et al. (2023) found that EIN3 and EIL1 interact with a histone demethylase to regulate FLOWERING LOCUS C and used etiolated hypocotyl assays to connect ethylene signaling activity with developmental transitions.
- Ban et al. (2025) demonstrated that BTB/POZ MATH proteins promote degradation of the Aux/IAA protein IAA10, revealing an auxin-controlled module that shapes *Arabidopsis* seedling development including hypocotyl elongation.

## Data Availability

No new data were generated or analyzed in support of this research. All information needed to evaluate the study is contained within the article.
